# A Neural Network-Enhanced Kalman Filter for Time Series Anomaly Detection in Cyber-Physical Systems

**DOI:** 10.3390/s26082332

**Published:** 2026-04-09

**Authors:** Zhongnan Ma, Wentao Xu, Hao Zhou, Ke Yu, Xiaofei Wu

**Affiliations:** School of Artificial Intelligence, Beijing University of Posts and Telecommunications, Beijing 100876, China; zhongnanma@bupt.edu.cn (Z.M.); xuwentao@bupt.edu.cn (W.X.); zhouh@bupt.edu.cn (H.Z.); wuxf@bupt.edu.cn (X.W.)

**Keywords:** anomaly detection, Kalman Filter, neural network, time series, Cyber-Physical Systems

## Abstract

Cyber-physical systems (CPSs) represent sophisticated intelligent architectures that tightly couple computational elements, communication networks, and physical processes. Their deployments now span virtually every industrial and civilian domain—from power grids and manufacturing plants to autonomous transportation networks. Ensuring the secure operation of CPSs relies fundamentally on effective time series anomaly detection, which remains a challenging task due to the complex, often unknown system dynamics and non-negligible sensor noise present in real-world environments. To address these challenges, we introduce a Neural Network-Enhanced Kalman Filter (NNEKF), a novel anomaly detection framework that combines model-based filtering with data-driven learning. The NNEKF employs a two-stage trained neural network with a specialized architecture: the first stage learns the underlying dynamics of the CPS, while the second stage optimizes the computation of the Kalman gain during the update step. At inference time, the enhanced Kalman filter recursively estimates the likelihood of observed sensor measurements to identify anomalies, supported by a batched parallel inference scheme that delivers substantial speedups. Extensive experiments on benchmark datasets demonstrate that the NNEKF attains an average F1-score of 0.935, coupled with rapid inference and minimal model footprint—surpassing all competitive baselines and facilitating dependable real-time anomaly detection for CPS environments.

## 1. Introduction

Cyber-physical systems (CPSs) integrate sensing and control functionalities with physical processes across diverse industrial domains, including smart grids, smart factories, and intelligent transportation systems [1,2]. These architectures depend on continuous, high-fidelity measurements to coordinate interconnected devices and process large-scale data streams. Yet their operational integrity is jeopardized by cyber-attacks, sensor faults, and human errors—risks that can corrupt measurement validity and inflict substantial economic or environmental damage. Safeguarding CPS security and data fidelity is therefore paramount. Effective sensor monitoring is essential, motivating the widespread adoption of time series anomaly detection to flag deviations in measurement streams. Whereas traditional statistical or rule-based methods falter amid the complexity of modern CPSs, deep learning-based techniques have gained traction as a compelling alternative.

Detecting anomalies in measurement signals remains an active research area. In particular, multivariate time series anomaly detection represents a core challenge for CPSs, with numerous methods proposed in the recent literature [3,4,5].

Conventional approaches encompass statistical methods, classical machine learning algorithms, and, increasingly, deep learning techniques. These data-driven methods have achieved remarkable progress, yet they often demand substantial training data and incur high time complexity.

In contrast, the Kalman filter (KF) offers inherent robustness against noise with low computational complexity, making it particularly suitable for resource-constrained CPS applications [6,7]. The KF operates through a recursive two-step process: in the prediction step, the prior state estimate is projected forward using a system dynamic model; in the update step, this prediction is refined by fusing the available observation with an optimally computed Kalman gain. This theoretically grounded framework has consistently demonstrated exceptional capability in estimating the states of dynamic systems.

Nevertheless, the KF’s efficacy hinges critically on accurate prior knowledge of system dynamics, typically encoded through a fully characterized state-space model. When the underlying physics are partially unknown or the system exhibits complex, nonlinear behaviors, the KF’s assumptions break down, leading to suboptimal estimation and degraded anomaly detection performance.

This fundamental limitation motivates our proposal of a hybrid approach that synergistically combines the theoretical soundness of the KF with the model-agnostic representation capabilities of deep neural networks (DNNs). The integration of the KF and DNN methodologies for time series anomaly detection presents several key challenges and research questions:

- **RQ1:** How can the state-space model be effectively learned from data?
- **RQ2:** How can we improve the calculation of Kalman gain?
- **RQ3:** What constitutes an appropriate evaluation metric for accurately distinguishing between normal and anomalous data points?

To address these questions, this paper proposes a novel time series anomaly detection framework for CPSs, termed the Neural Network-Enhanced Kalman Filter (NNEKF). Specifically, we employ a two-stage trained neural network with a specialized architecture. In the first stage, the neural network learns the state transition function of the CPS, thereby capturing its underlying dynamics. In the second stage, the network optimizes the calculation of the Kalman gain in the update step. During the detection phase, the refined KF is naturally applied to track the uncertainty of the hidden states of the system and estimate the likelihood of the observed sensor measurements over time. The NNEKF framework integrates the strengths of both the KF and neural networks for time series anomaly detection. As a result, the NNEKF benefits from the ability to capture highly nonlinear system dynamics while maintaining robustness against process and sensor noises inherent in CPSs. We summarize the main contributions of our paper as follows:

- We propose a novel anomaly detection architecture that integrates Kalman filtering with deep learning. A two-stage trained neural network first encodes system dynamics through the state transition function, then adaptively refines Kalman gain computation. The enhanced filter recursively evaluates measurement likelihoods for efficient anomaly identification.
- We introduce a batched parallel inference mechanism that delivers orders-of-magnitude inference acceleration, achieving fast response time and low memory footprint—key requirements for real-time, resource-constrained CPS deployments.
- Comprehensive experiments demonstrate that our method achieves an average F1-score of 0.935, comparable to state-of-the-art approaches in CPS anomaly detection, while maintaining low inference latency.
- Ablation studies validate the efficacy of core architectural components, including the attention mechanism, *K*-network, and two-stage training. We further analyze the model’s sensitivity to key hyperparameters, noise, and missing data.

## 2. Background

### 2.1. Cyber-Physical Systems

**Figure 1:**
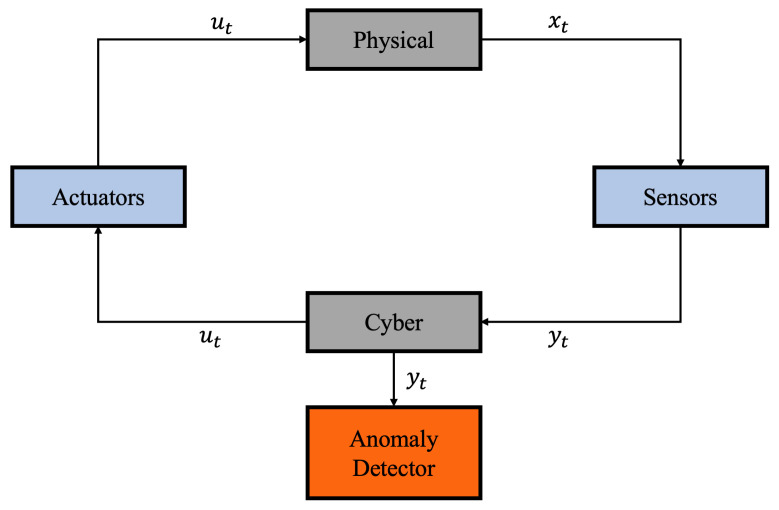
Anomaly detection architecture in CPSs.

In CPSs, the physical domain encompasses real-world processes requiring supervision, while the cyber domain comprises communication, computation, and control functionalities. These layers interface through sensors and actuators: sensors acquire the system state $x_{t}$ and transduce it into observations $y_{t}$, while the cyber layer and actuators generate control inputs $u_{t}$. For anomaly detection, $y_{t}$ is analyzed to estimate its probability distribution. An alert is triggered when the residual error surpasses a predefined threshold or the measurement likelihood drops below a specified bound [8], as illustrated in Figure 1.

The CPS can be typically characterized by a discrete-time nonlinear state-space representation, comprising two fundamental components: a state transition model and a measurement model.

State Transition Model: Consider a hidden state vector $x_{t}\in R^{n}$ at time step *t*. The state evolution is governed by the following:

(1) $$x_{t}=f\left(x_{t-1},u_{t}\right)+w_{t},\;w_{t}∼N\left(0,Q\right),$$

where the state transition incorporates both deterministic dynamics and stochastic perturbations. The deterministic component is described by a nonlinear mapping $f:R^{n}\times R^{m}\to R^{n}$, with $x_{t}\in R^{n}$ representing the system state and $u_{t}\in R^{m}$ denoting the control input. The stochastic component $w_{t}$ represents zero-mean Gaussian process noise with covariance matrix Q.

Measurement Model: The observation process is modeled as follows:

(2) $$y_{t}=h\left(x_{t}\right)+v_{t},\;v_{t}∼N\left(0,R\right),$$

where $y_{t}\in R^{p}$ constitutes the observation vector, $h:R^{n}\to R^{p}$ is the measurement mapping, and $v_{t}$ represents zero-mean Gaussian measurement noise with covariance matrix R. At each time *t*, the observed measurement $y_{t}$ is a noise-corrupted version of the true system state.

### 2.2. Kalman Filter

Our proposed model builds upon the classical KF framework, necessitating a brief overview. The KF is an optimal linear recursive estimator that minimizes the mean squared error under Gaussian noise assumptions. For linear systems, there exist matrices F (state transition), B (control input), and H (observation) such that

(3) $$f\left(x_{t-1},u_{t}\right)={\mathrm{Fx}}_{t-1}+{\mathrm{Bu}}_{t},\;h\left(x_{t}\right)={\mathrm{Hx}}_{t}$$

The KF operates in two phases:

predict:

(4a) $${\hat{x}}_{t|t-1}={\mathrm{Fx}}_{t-1}+{\mathrm{Bu}}_{t},$$

(4b) $${\hat{\Sigma }}_{t|t-1}=F\Sigma_{t-1}F^{T}+Q.$$

(5a) $${\hat{y}}_{t|t-1}=H{\hat{x}}_{t|t-1},$$

(5b) $$S_{t}=H{\hat{\Sigma }}_{t|t-1}H^{T}+R.$$

update:

(6a) $${\hat{x}}_{t}={\hat{x}}_{t|t-1}+K_{t}▵y_{t},$$

(6b) $${\hat{\Sigma }}_{t}=\left(I-K_{t}H_{t}\right){\hat{\Sigma }}_{t|t-1}.$$

The Kalman gain matrix $K_{t}$ is computed as follows:

(7) $$K_{t}={\hat{\Sigma }}_{t|t-1}H^{T}S_{t}^{-1},$$

with innovation $▵y_{t}=y_{t}-{\hat{y}}_{t|t-1}$.

The Extended Kalman Filter (EKF) [9] generalizes the KF to nonlinear systems via first-order Taylor linearization:

(8) $${\hat{x}}_{t|t-1}=f\left(x_{t-1},u_{t-1}\right),\;F_{t}=J_{f}\left(x_{t-1}\right)$$

(9) $${\hat{y}}_{t|t-1}=h\left({\hat{x}}_{t|t-1}\right),\;H_{t}=J_{h}\left({\hat{x}}_{t|t-1}\right)$$

where $F_{t}$ and $H_{t}$ are the Jacobians used in covariance propagation and gain computation.

### 2.3. Related Work

Time series anomaly detection has witnessed substantial progress through deep learning methodologies.

Reconstruction-based architectures have demonstrated particular effectiveness for this task. The Deep Autoencoding Gaussian Mixture Model (DAGMM) [10] integrates deep autoencoders with Gaussian Mixture Models (GMMs) for latent space probabilistic modeling. OmniAnomaly [11] employs Gated Recurrent Units (GRUs) and Variational Autoencoders (VAEs) to identify anomalies based on reconstruction probabilities. USAD [12] introduces an unsupervised adversarial framework for multivariate time series, while GDN [13] leverages graph neural networks to capture sensor dependencies for anomaly detection. DTGMM [14] combines deep autoencoders, Transformer, and GMMs to enhance condition monitoring and early warning capabilities for critical equipment such as boiler superheaters and turbine bearings.

Attention mechanisms have emerged as a critical component for enhancing temporal modeling in anomaly detection. Anomaly Transformer [15] utilizes self-attention with a minimax training strategy to amplify discrepancies between normal and abnormal patterns. Beyond time series anomaly detection, attention–GRU architectures have demonstrated superior performance over traditional methods in industrial predictive maintenance scenarios [16]. These developments corroborate our motivation for employing attention mechanisms to refine state transition learning within a Kalman filtering framework.

Concurrently, model-based techniques—particularly Kalman filter variants—have gained renewed attention for anomaly detection in dynamical systems. The standard KF assumes linear dynamics and Gaussian noise, which often prove inadequate in practice. To address nonlinearities, the Extended Kalman Filter (EKF) [9] employs first-order Taylor expansion (Equations (8) and (9)), while the unscented Kalman filter (UKF) [17] utilizes deterministic sampling for improved state estimation. The Particle Filter (PF) [18] accommodates non-Gaussian distributions through sequential Monte Carlo methods. However, these extensions fundamentally rely on explicit system models, limiting applicability when dynamics are unknown or evolve over time.

Two emerging research directions have sought to integrate deep learning with Kalman filtering, directly motivating our work on state-space model learning (**RQ1**) and Kalman gain learning (**RQ2**).

**Learnable Kalman Gain (RQ2).** The conventional Kalman gain is theoretically optimal only for linear Gaussian systems; under nonlinear or non-Gaussian conditions, it becomes suboptimal and degrades tracking performance over time. To address this, KalmanNet [19] and Split-KalmanNet [20] employ GRUs to learn the gain directly from observation-state residuals, bypassing explicit covariance calculations. While these approaches demonstrate that neural augmentation in the update step improves filtering performance, they do not address the simultaneous learning of system dynamics.

**Neural State-Space Models (RQ1).** For systems with complex or partially known dynamics, an alternative strategy encodes observations into a latent space governed by a simplified (typically linear Gaussian) state-space model [21,22,23]. Complementing this, refs. [24,25,26] directly estimate state-space parameters via neural networks to mitigate model mismatch—a critical limitation of conventional KF requiring precise model specifications.

Our work advances neural-augmented Kalman filtering for anomaly detection. While NSIBF [27] learns state transition and measurement functions, it fails to capture long-range dependencies and incurs high inference costs. We address these limitations through: (1) an attention mechanism for refined state transition learning (**RQ1**), inspired by attention–GRU architectures [16], and (2) a lightweight neural network for dynamic Kalman gain computation (**RQ2**) with parallel inference for significant speedup. This integration achieves superior accuracy with lower computational overhead than prior approaches.

## 3. Methodology

This section presents our proposed neural network architecture for state-space model learning, Kalman gain learning, and the integrated NNEKF framework for time series anomaly detection.

### 3.1. State-Space Model Learning

**Figure 2:**
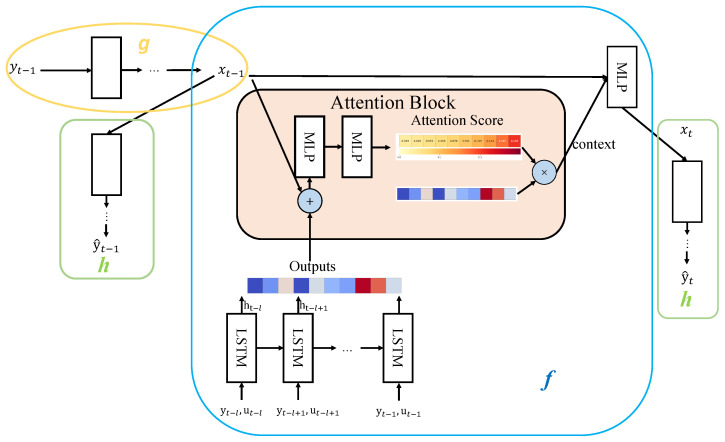
Neural network architecture for state-space model learning. Blue, yellow, and green boxes represent networks *f*, *g*, and *h*, respectively.

Let $x_{t}$, $y_{t}$, and $u_{t}$ denote the hidden state, sensor observation, and actuator state, respectively, at discrete time *t*. To address **RQ1**, we propose a neural network architecture that employs self-attention mechanisms to learn the underlying state-space dynamics. As illustrated in Figure 2, our framework comprises three subnets (*f*, *g*, and *h*):

- The network *f* learns the state transition function. It operates on two inputs: the current hidden state $x_{t-1}$ and a historical sequence ${\left(y,u\right)}^{t-l:t-1}$ of sensor observations and actuator states from a sliding window of length *l*.
  The historical sequence is first encoded by LSTM layers [28] into hidden representations $\left\{h_{t-l},\ldots ,h_{t-1}\right\}$. Unlike prior work such as NSIBF [27] that relies solely on LSTM for temporal modeling, we employ a self-attention mechanism where $x_{t-1}$ serves as the query and each LSTM output $h_{i}$ as key–value pairs. Attention weights $\alpha_{i}=\mathrm{softmax}\left(a_{i}\right)$ are computed via learned compatibility functions, allowing for dynamic focus on relevant historical moments. The context vector $c_{t-1}=\sum_{i=t-l}^{t-1}\alpha_{i}h_{i}$ is concatenated with $x_{t-1}$ and mapped to $x_{t}$ through an MLP.
- The network *g* encodes the sensor observation $y_{t-1}$ into the corresponding hidden state $x_{t-1}$.
- The network *h* reconstructs the sensor observation ${\tilde{y}}_{t-1}$ from hidden state $x_{t-1}$.

The network accepts $y_{t-1}$ and historical sequence ${\left(y,u\right)}^{t-l:t-1}$ as inputs, producing reconstructed observation ${\tilde{y}}_{t-1}$ and predicted observation ${\tilde{y}}_{t}$ as outputs.

Assume the training dataset comprises *T* time points. The loss function is as follows:

(10) $$L=\sum_{t=l}^{T}(w_{1}∥y^{t-1}-{\tilde{y}}^{t-1}{∥}_{2}^{2}+w_{2}∥y^{t}-{\tilde{y}}^{t}{∥}_{2}^{2}+w_{3}∥x^{t}-x^{t-1}{∥}_{2}^{2})$$

where the first two terms capture reconstruction and prediction errors, the third term enforces temporal smoothness, and $w_{1}$, $w_{2}$, and $w_{3}$ denote weighting hyperparameters. We set $w_{1}=0.45$, $w_{2}=0.45$, and $w_{3}=0.1$, yielding a combined weight of 0.9 for the reconstruction and prediction terms. This configuration ensures the accurate modeling of system observations, while the relatively small weight on smoothness prevents over-regularization and preserves responsiveness to rapid state changes. The detailed hyperparameter configuration is provided in Appendix A.

After training, we obtain the learned state transition and measurement functions:

(11) $$x_{t}=f\left(x_{t-1},\left\{y_{t-l:t-1},u_{t-l:t-1}\right\}\right),y_{t}=h\left(x_{t}\right)$$

These correspond to the functions f(·) and h(·) defined in Equations (1) and (2), respectively.

### 3.2. Kalman Gain Learning

#### 3.2.1. Overall Architecture

**Figure 3:**
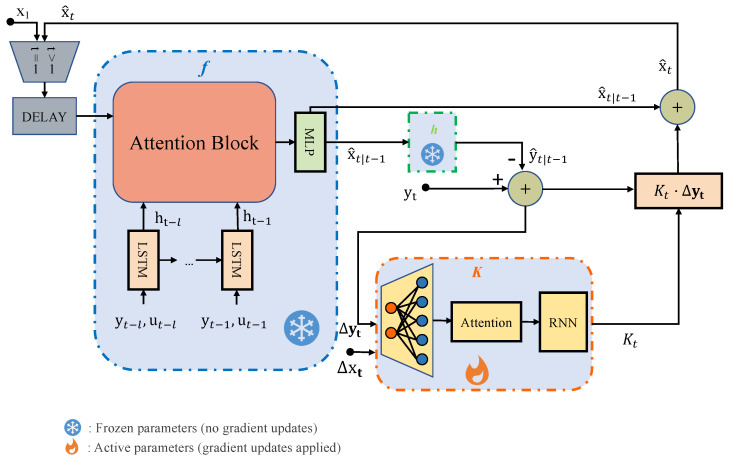
The overall architecture of the NNEKF. Parameters of networks *f* and *g* are frozen, while parameters of network *K* are active (trainable).

To address **RQ2**, we present an enhanced KF algorithm in this section. Specifically, we introduce a *K*-network to learn the Kalman gain (KG) from data, which is then integrated with the previously learned system dynamics f(·) and measurement functions h(·) from Section 3.1 within the comprehensive KF framework. The overall architecture of our proposed approach is summarized in Figure 3. In each time instance *t*, similarly to the KF, the forward propagation of the NNEKF is divided into two steps: predict and update. The difference is that the NNEKF only keeps track of the mean estimation and does not track covariance estimation.

predict: The prior state estimate ${\hat{x}}_{t|t-1}$ is computed using the posterior estimate from the previous time step ${\hat{x}}_{t-1}$ along with sensor measurements and actuator states from a sliding window of the past *l* time steps $W_{t-1}=\left\{y_{t-l:t-1},u_{t-l:t-1}\right\}$, as defined in (12a). Note that the initial posterior estimate ${\hat{x}}_{1}$ is derived from the initial observation $y_{1}$ via Equation (12c).

Subsequently, the prior observation estimate ${\hat{y}}_{t|t-1}$ is derived from ${\hat{x}}_{t|t-1}$ through (12b). Both f(·) and h(·) are learned as described in Section 3.1.

(12a) $${\hat{x}}_{t|t-1}=f\left({\hat{x}}_{t-1},W_{t-1}\right),$$

(12b) $${\hat{y}}_{t|t-1}=h({\hat{x}}_{t|t-1}).$$

(12c) $${\hat{x}}_{1}=h\left(y_{1}\right)$$

update: The NNEKF updates the posterior state estimate ${\hat{x}}_{t}$ using the new observation $y_{t}$ and prior estimate ${\hat{x}}_{t|t-1}$ through a process similar to the classical KF (Equations (13a) and (13b)). However, unlike the conventional KF, the KG is not computed analytically but learned directly from data using an RNN (13c). The recurrent architecture’s inherent memory enables implicit KG computation without explicit covariance statistics.

(13a) $$▵y_{t}=y_{t}-{\hat{y}}_{t|t-1},$$

(13b) $${\hat{x}}_{t}={\hat{x}}_{t|t-1}+K_{t}▵y_{t},$$

(13c) $$K_{t}=K\left(▵y_{t},▵{\hat{y}}_{t},▵{\hat{x}}_{t},▵x_{t}\right).$$

#### 3.2.2. *K*-Network Architecture

In the following, we detail the design of the *K*-network for learning the Kalman gain (KG). The computation of KG requires inputs that capture the statistical relationships between observations and state estimates. At each time step *t*, the *K*-network receives inputs that encode the statistical properties of both the current observation $y_{t}$ and the previous state estimate ${\hat{x}}_{t-1}$.

We define the following input features to characterize the unknown statistical relationships within the CPS model:

- F1: Observation difference $▵y_{t}=y_{t}-y_{t-1}$.
- F2: Innovation difference $▵{\hat{y}}_{t}=y_{t}-{\hat{y}}_{t|t-1}$.
- F3: State posterior difference $▵{\hat{x}}_{t}={\hat{x}}_{t}-{\hat{x}}_{t-1}$.
- F4: State update difference $▵x_{t}={\hat{x}}_{t}-{\hat{x}}_{t|t-1}$.

These four features are designed to enable the *K*-network to learn the underlying statistics of states and observations effectively, thereby supporting accurate KG computation.

**Figure 4:**
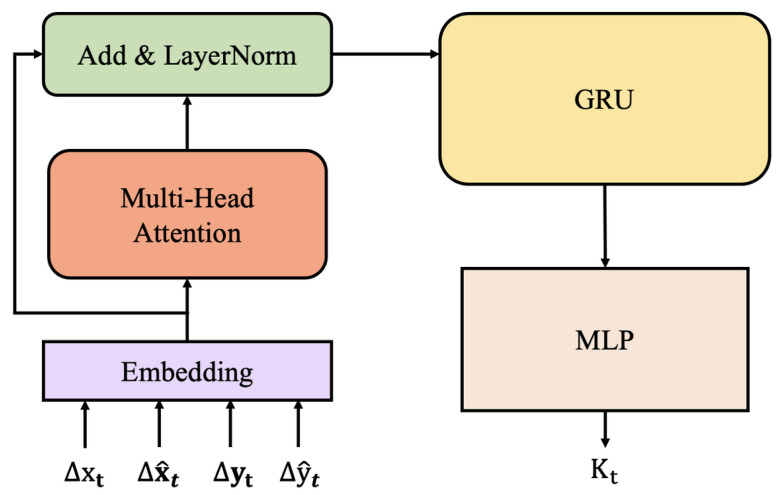
The architecture of the *K*-network with an attention mechanism.

We design the *K*-network using attention mechanisms and Gated Recurrent Units (GRUs). As shown in Figure 4, it integrates multi-head attention with GRU-based memory to model statistical dependencies for Kalman gain estimation. Input features are first embedded into a latent space, then processed by multi-head attention with residual connections and layer normalization. The refined features pass through a GRU to capture temporal dynamics in hidden state $h_{t}$, which is decoded into Kalman gain $K_{t}\in R^{m\times n}$ via a linear output layer.

We define the MSE loss between predicted and actual observations:

(14) $$L=∥{\hat{y}}_{t}-y_{t}{∥}^{2}.$$

(15) $${\hat{y}}_{t}=h\left({\hat{x}}_{t}\right)$$

We partition the training dataset into *N* distinct sequences. Specifically, denoting the length of the *i*-th training sequence as $T_{i}$, the dataset can be formally represented as $D={\left\{Y_{i}\right\}}_{i=1}^{N}$, where $Y_{i}=\left[y_{1}^{i},y_{2}^{i},\ldots ,y_{T_{i}}^{i}\right]$ represents the *i*-th sequence.

Letting θ denote the trainable parameters of the *K*-network, we formulate the mean squared error (MSE) loss function as follows:

(16) $$L\left(\theta \right)=\frac{1}{N}\sum_{i=1}^{N}\sum_{t=1}^{T_{i}}{∥{\hat{y}}_{t}^{i}-y_{t}^{i}∥}^{2},$$

where ${\hat{y}}_{t}^{i}$ denotes the predicted observation at time step *t* for the *i*-th sequence.

While forward propagation requires the complete NNEKF architecture (including *f*-net and *h*-net), we focus exclusively on KG learning during backpropagation. Consequently, we freeze the pre-trained parameters of *f*-net and *h*-net, updating only the *K*-network’s parameters.

### 3.3. Analysis of Learning Algorithm

This subsection analyzes the gradient propagation mechanism to elucidate why end-to-end training suffers from severe gradient interference and how the proposed two-stage training achieves gradient decoupling to ensure stable convergence.

Let $\theta_{dyn}=\left\{\theta_{f},\theta_{g},\theta_{h}\right\}$ denote the parameters of the state-space model learning module (comprising networks *f*, *g*, and *h*), and let $\theta_{kg}$ denote the parameters of the Kalman gain network $K_{\theta_{kg}}$. The dynamic learning loss $L_{dyn}\left(\theta_{dyn}\right)$ and the Kalman gain learning loss $L_{kg}\left(\theta_{dyn},\theta_{kg}\right)$ are defined in Equations (10) and (14), respectively.

#### 3.3.1. Gradient Interference in End-to-End Training

Under end-to-end joint training, the total loss is $L_{dyn}\left(\theta_{dyn}\right)$, which is defined in

(17) $$L_{total}\left(\theta_{dyn},\theta_{kg}\right)=L_{dyn}\left(\theta_{dyn}\right)+\lambda L_{kg}\left(\theta_{dyn},\theta_{kg}\right),\;\lambda >0.$$

The gradient with respect to $\theta_{dyn}$ contains a destructive cross-term:

(18) $$\nabla_{\theta_{dyn}}L_{total}=\nabla_{\theta_{dyn}}L_{dyn}+\lambda \nabla_{\theta_{dyn}}L_{kg},$$

where the cross-term expands to the following:

(19) $$\nabla_{\theta_{dyn}}L_{kg}=\sum_{t=1}^{T}2{\left(y_{t}-{\hat{y}}_{t|t}\right)}^{⊤}\frac{\partial h({\hat{x}}_{t|t})}{\partial {\hat{x}}_{t|t}}\left(\frac{\partial {\hat{x}}_{t|t-1}}{\partial \theta_{dyn}}+K_{t}\frac{\partial (y_{t}-h\left({\hat{x}}_{t|t-1}\right))}{\partial \theta_{dyn}}\right).$$

Because the Kalman filter is recursive, gradient conflicts accumulate over time steps, progressively amplifying modeling errors in the dynamic module. This mirrors the gradient pathology in Physics-Informed Neural Networks (PINNs), where the simultaneous optimization of competing objectives leads to destructive interference [29]. Specifically,

- $\nabla_{\theta_{dyn}}L_{dyn}$ pushes $\theta_{dyn}$ toward stable, physically consistent state transitions;
- $\nabla_{\theta_{dyn}}L_{kg}$ adapts $\theta_{dyn}$ to compensate for instantaneous Kalman gain errors, encouraging overfitting to closed-loop residuals.

Simultaneously optimizing these incompatible objectives causes training instability, slow convergence, and poor generalization—a failure mode analogous to PINN training collapse in convection-dominated PDEs [29].

#### 3.3.2. Gradient Decoupling via Two-Stage Training

The proposed two-stage training eliminates interference by sequential optimization with parameter freezing.

Stage 1: State-Space Model Learning. Only the dynamic loss $L_{dyn}$ is optimized:

$$\theta_{dyn}^{★}=\arg\min_{\theta_{dyn}}L_{dyn}\left(\theta_{dyn}\right).$$

The Kalman gain network is inactive, so the gradient updates for $\theta_{dyn}$ are guided solely by reconstruction and prediction errors. This ensures that the dynamic module converges to a physically consistent representation of the CPS dynamics, unaffected by the filtering objective.

Stage 2: Kalman Gain Learning. After Stage 1, $\theta_{dyn}^{★}$ is frozen. As a result, the cross-term $\nabla_{\theta_{dyn}}L_{kg}$ vanishes, and the gradient of $L_{kg}$ no longer propagates into the dynamic module. The Kalman gain network is optimized independently within a fixed, stable feature space, completely decoupled from the dynamic model. Both sub-tasks therefore converge along their own optimal directions, free from conflicting gradient signals.

### 3.4. Anomaly Detection

In this section, we address anomaly detection in multivariate time series observations $y_{t}$. Let $y_{t}\in R^{m}$ denote the *m*-dimensional observation vector at time *t*, where each component $y_{t,i}$ represents the *i*-th variable. An anomaly score $S_{t}$ is computed at each time step, and an observation is flagged as anomalous if $S_{t}$ exceeds a predefined threshold.

The anomaly score $S_{t}$ for observation $y_{t}$ requires an estimate ${\hat{y}}_{t}$ of the expected observation, obtained through the following recursive procedure:

1. Initialization: ${\hat{x}}_{1}=h\left(y_{1}\right)$.
2. Prediction: Compute the prior state estimate ${\hat{x}}_{t|t-1}$ via (12a) and the prior observation estimate ${\hat{y}}_{t|t-1}$ via (12b).
3. Update: Obtain the posterior state estimate ${\hat{x}}_{t}$ via (13a)–(13c), and compute the posterior observation estimate ${\hat{y}}_{t}$ via (15).

The posterior state estimate ${\hat{x}}_{t}$ is then used to predict the next prior state ${\hat{x}}_{t+1|t}$ and observation ${\hat{y}}_{t+1|t}$. The estimated observation ${\hat{y}}_{t}$ serves to compute the anomaly score $S_{t}$ for the current observation $y_{t}$.

To address **RQ3**, an appropriate anomaly metric is crucial for effective detection. A straightforward approach computes the Euclidean distance between $y_{t}$ and ${\hat{y}}_{t}$, equivalent to the loss function in (14):

(20) $$S_{t}={∥{\hat{y}}_{t}-y_{t}∥}^{2}.$$

Alternatively, following NSIBF [27], we employ the Mahalanobis distance (MD) [30]:

(21) $$S_{t}=\sqrt{{\left(y_{t}-{\hat{y}}_{t}\right)}^{⊤}R^{-1}\left(y_{t}-{\hat{y}}_{t}\right)}.$$

We introduce the covariance matrix R to capture observation uncertainty, estimated as follows:

(22a) $${\tilde{y}}_{t}=h\left(x_{t}\right),\;\Delta {\tilde{y}}_{t}={\tilde{y}}_{t}-y_{t},$$

(22b) $$\Delta \tilde{Y}={\left[\Delta {\tilde{y}}_{1},\ldots ,\Delta {\tilde{y}}_{T}\right]}^{⊤}\in R^{T\times m},$$

(22c) $$R=\mathrm{cov}\left(\Delta \tilde{Y}\right)\in R^{m\times m},$$

where $\Delta {\tilde{y}}_{t}$ represents the reconstruction error at time *t*, and R provides a global estimate of observation uncertainty across the training dataset of size *T*.

As detailed in Algorithm 1, the per-step time complexity of the NNEKF is $O(ln^{2}+nm+m^{2})$, with *l* denoting the window length, *n* the state dimension, and *m* the observation dimension. By comparison, the standard Kalman filter requires $O(n^{3})$ operations stemming from explicit covariance propagation in (7) and the update step. These complexities align when *n* and *m* are comparable in scale.
**Algorithm 1** NNEKF Inference (Anomaly Detection) at Time Step *t***Require:** Posterior state estimate ${\hat{x}}_{t-1}$, historical window $W_{t-1}=\left\{y_{t-l:t-1},u_{t-l:t-1}\right\}$, current observation $y_{t}$, pre-trained networks *f*, *h*, *K*, covariance matrix R (estimated from training data).**Ensure**: Posterior state estimate ${\hat{x}}_{t}$, anomaly score $S_{t}$.  1: **Predict step**  2: ${\hat{x}}_{t|t-1}\leftarrow f\left({\hat{x}}_{t-1},W_{t-1}\right)$▹$O(l\cdot n^{2})$  3: ${\hat{y}}_{t|t-1}\leftarrow h\left({\hat{x}}_{t|t-1}\right)$▹O(n·m)  4: **Compute innovation and features**  5: $\Delta y_{t}\leftarrow y_{t}-{\hat{y}}_{t|t-1}$▹O(m)  6: $\Delta {\hat{y}}_{t}\leftarrow y_{t}-{\hat{y}}_{t|t-1}$▹O(m)  7: $\Delta {\hat{x}}_{t}\leftarrow {\hat{x}}_{t}-{\hat{x}}_{t-1}$▹O(n)  8: $\Delta x_{t}\leftarrow {\hat{x}}_{t}-{\hat{x}}_{t|t-1}$▹O(n)  9: **Kalman gain computation**10: $K_{t}\leftarrow K\left(\Delta y_{t},\Delta {\hat{y}}_{t},\Delta {\hat{x}}_{t},\Delta x_{t}\right)$▹O(n·m)11: **Update step**12: ${\hat{x}}_{t}\leftarrow {\hat{x}}_{t|t-1}+K_{t}\;\Delta y_{t}$▹O(n·m)13: **Compute anomaly score**14: **if** using MSE **then**15:     $S_{t}\leftarrow {∥y_{t}-{\hat{y}}_{t|t-1}∥}^{2}$▹O(m)16: **else**17:     $S_{t}\leftarrow \sqrt{{\left(y_{t}-{\hat{y}}_{t|t-1}\right)}^{⊤}R^{-1}\left(y_{t}-{\hat{y}}_{t|t-1}\right)}$▹$O(m^{2})$18: **end if**19: **return**
${\hat{x}}_{t}$, $S_{t}$

The baseline NSIBF [27] employs an unscented Kalman filter (UKF) built upon deterministic sigma points. In practice, its runtime substantially surpasses $O(n^{3})$ owing to the overhead of propagating 2n+1 sigma points through nonlinear transformations. Furthermore, traditional Kalman-type estimators—including NSIBF—adhere to a rigid sequential structure: the estimate ${\hat{x}}_{t}$ is contingent upon ${\hat{x}}_{t-1}$, precluding straightforward parallelization across time steps.

**Figure 5:**
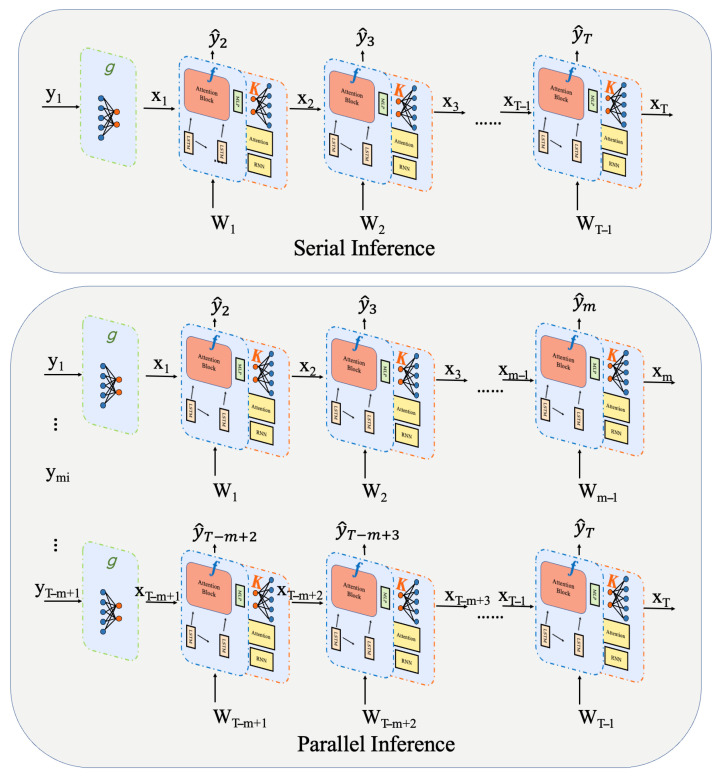
Serial inference versus batched parallel inference.

The NNEKF circumvents this constraint by exploiting the observation mapping h(·) to initialize states at arbitrary time steps, thereby severing long-range temporal dependencies. This facilitates the batched parallel inference framework depicted in Figure 5: the time series is segmented into *B* autonomous batches, each initialized by applying h(·) to its leading observation. All batches undergo simultaneous GPU processing following the identical recursive update rules as sequential execution, with anomaly scores subsequently concatenated. This parallel paradigm curtails wall-clock time to roughly $O\left(\left(T/B\right)\cdot \left(ln^{2}+nm+m^{2}\right)\right)$, as corroborated empirically in Section 4.4, where the NNEKF delivers orders-of-magnitude acceleration relative to NSIBF across all four datasets.

## 4. Experiments and Results

In this section, we evaluate our proposed method for anomaly detection on four real-world CPS datasets and compare the performance with several competitive anomaly detection methods.

### 4.1. Datasets and Baselines

We consider the following four real-world CPS datasets:

- ASD [31]: Twelve server entities with 19 metrics (CPU, memory, network, VM, etc.) at 5 min intervals, with expert-labeled anomalies.
- SMD [11]: Twelve machine entities with 38 metrics at 1 min intervals.
- PUMP [27]: Water pump system data at 1 min granularity over five months.
- SMAP [32]: Soil and telemetry data from NASA’s Mars rover.

More specifications of our datasets are given in Table 1.

We compare the NNEKF against several established anomaly detection methods:

- iForest [33]: A tree-based method detecting anomalies via recursive data partitioning.
- DAGMM [10]: Combines deep autoencoders with Gaussian Mixture Models for latent space modeling.
- OmniAnomaly [11]: A deep generative model using GRU-VAE with normalizing flows, employing reconstruction probabilities as anomaly scores.
- AnomalyTrans [15]: Uses an anomaly–attention mechanism to compute association discrepancy with a minimax strategy.
- DCdetector [34]: Employs dual-attention asymmetric design with contrastive learning for permutation-invariant representations.
- DADA [35]: Uses adaptive bottlenecks for dynamic temporal compression and dual adversarial decoders to amplify deviations.
- KAN-AD [36]: Replaces MLPs with Kolmogorov–Arnold Networks to capture nonlinear dependencies via decomposed univariate functions.
- NSIBF [27]: Learns CPS dynamics via neural networks followed by Kalman filter state tracking.

DAGMM and OmniAnomaly represent early deep learning approaches, while AnomalyTrans, DCdetector, and DADA represent recent advances. KAN-AD achieves state-of-the-art performance by leveraging Kolmogorov–Arnold Networks for nonlinear temporal modeling. NSIBF serves as a crucial baseline combining neural networks with Kalman filtering, enabling direct comparison with our Neural Network-Enhanced Kalman Filter.

To evaluate the effects of two distinct anomaly scores—minimum squared error (MSE) and Mahalanobis distance (MD)—we introduce two corresponding models: NNEKF-MSE and NNEKF-MD. The former computes the MSE between the estimated observation and the ground truth, while the latter computes the Mahalanobis distance using a covariance matrix R.

### 4.2. Performance and Analysis

We adopt precision, recall, and F1-score as evaluation metrics, with particular emphasis on F1 due to its balanced trade-off. Table 2 and Table 3 summarize the performance (mean ± half-width of 95% confidence interval) for all methods and datasets.

KAN-AD achieves the best overall performance with the highest average F1-score (0.938). Our proposed NNEKF-MD and NNEKF-MSE rank second (0.935) and third (0.933), respectively. NSIBF exhibits consistent performance with the fourth best mean F1-score (0.918), which both NNEKF variants outperform.

Among baselines, KAN-AD and AnomalyTrans perform strongly on SMD, PUMP, and SMAP but are degraded markedly on ASD—a dataset with limited training data (approximately 8000 samples per subset). This suggests that these methods may struggle in data-scarce scenarios.

To validate robustness and efficiency, Table 3 reports the mean ± half-width of the 95% confidence interval for average F1-scores and inference times over five independent runs. Although KAN-AD achieves the highest average F1-score, our NNEKF model ranks second while delivering the fastest inference time, enabled by our batched parallel inference strategy. Notably, this parallel implementation reduces inference time by over two orders of magnitude compared to NSIBF without sacrificing accuracy. This speedup stems from the batched parallel inference strategy introduced in Section 3.4, which eliminates the sequential bottleneck inherent in traditional Kalman filters.

A detailed cost analysis is provided in Table 4 and Table 5. NSIBF requires 680–7300 s for combined training and inference across datasets, with 0.43–1.83 MB storage.

The NNEKF employs two-stage training: Stage 1 learns state-space dynamics (*f*-net and *h*-net); Stage 2 refines the Kalman gain network (*K*-net) while freezing the dynamic modules. Although this extends training marginally, the NNEKF achieves substantially faster inference by replacing NSIBF’s sigma point sampling with parallelized batch processing. With 32 batches applied uniformly across all datasets, our method achieves 3.1× to 22.3× speedups over NSIBF and requires only 0.80–3.22 MB of storage, making it well-suited for resource-constrained CPS environments.

**Figure 6:**
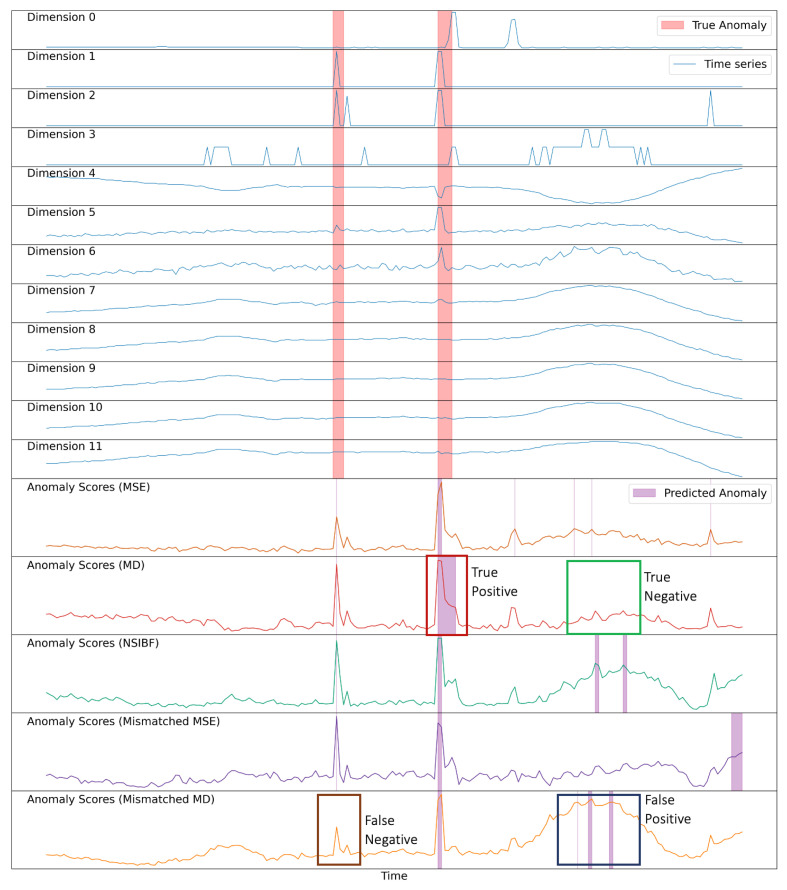
Visualization of anomaly scores for ASD data segments, with anomaly scores from NNEKF-MSE, NNEKF-MD, and NSIBF.

To better understand the NNEKF’s behavior, we examine its anomaly scores on ASD. Figure 6 presents the scores generated by NNEKF-MD, NNEKF-MSE, and NSIBF for segments from the ASD dataset. Red-highlighted regions denote true anomalies; purple-highlighted regions indicate predicted anomalies. While all three models produce elevated scores within anomaly regions, NNEKF-MSE exhibits irregular fluctuations due to noise sensitivity, and NSIBF suffers from false-positive fluctuations that degrade precision. In contrast, NNEKF-MD demonstrates the most reliable behavior: stable scores in normal regions with elevation confined to anomalous regions. This explains NNEKF-MD’s superior F1-score on ASD.

The superior performance of NNEKF-MD stems from its use of Mahalanobis distance, which incorporates the covariance matrix R to decorrelate features. However, this reliance reveals a key limitation: R is precomputed from training residuals (Equation (22)) and thus depends heavily on the accuracy of the learned state-space model. When the model accurately captures system dynamics, R effectively enhances detection; otherwise, estimation errors propagate into the anomaly score.

To simulate this scenario, we deliberately compromise state-space model learning by selecting improper loss weights $w_{1}=0.3$; $w_{2}=0.1$; $w_{3}=0.6$ (Equation (10)), compared to the proper configuration ($w_{1}=0.45$; $w_{2}=0.45$; $w_{3}=0.1$). This impairs *f*-net training, causing model mismatch. Figure 6 shows anomaly scores under this mismatch: both the MSE and MD produce compromised predictions, with MD exhibiting worse degradation—false negatives in anomalous regions and false-positive fluctuations in normal regions. This confirms that NNEKF-MD performance critically depends on state-space model accuracy.

A more principled alternative would be to estimate $R_{t}$ online within the *K*-network, but this would substantially increase complexity and inference latency. Given our goal of balancing accuracy, speed, and model footprint for real-time CPS deployments, we adopted the lightweight precomputation strategy.

### 4.3. Ablation Study

**Figure 7:**
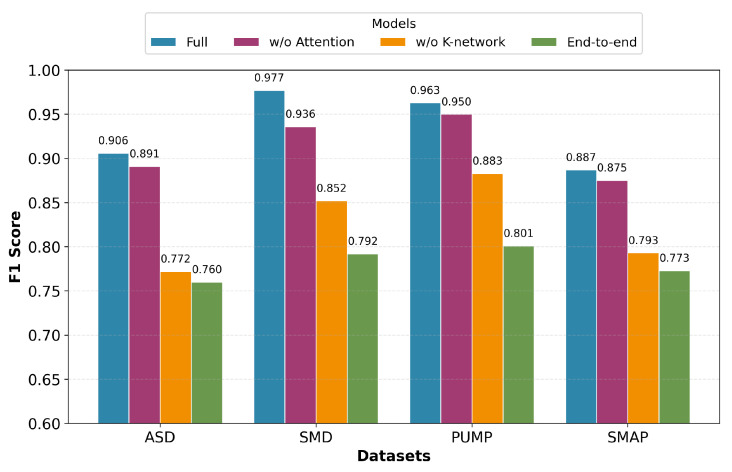
An F1-score comparison of the full NNEKF model against its ablated variants across three datasets.

To evaluate the core contributions of the NNEKF, we conduct ablation studies on (1) the attention mechanism in state-space modeling, (2) the learnable *K*-network for Kalman gain, and (3) end-to-end versus two-stage training. We compare the full model against three ablated variants (Figure 7), each employing the MSE-based anomaly score:

- Full Model: The complete NNEKF framework employing attention-based state transition learning, the dedicated *K*-network, and two-stage training.
- w/o Attention: Replaces the attention mechanism with a standard LSTM in the state-space module to isolate its contribution to temporal dynamic modeling.
- w/o K-Network (EKF): Substitutes the learned *K*-network with the analytical Extended Kalman Filter gain to validate the necessity of data-driven gain learning.
- End-to-End: Jointly optimizes the state-space model and *K*-network via Equation (17) (λ=1.0), eliminating the two-stage training procedure.

As shown in Figure 7, the full model consistently outperforms all variants. The w/o Attention variant exhibits degraded F1-scores, confirming that attention is crucial for capturing complex temporal dependencies. The w/o K-Network variant shows the most significant performance drop, empirically validating that data-driven Kalman gain is essential for optimal state estimation in nonlinear CPS environments. Notably, the End-to-End variant performs the worst across all datasets, corroborating our analysis in Section 3.3 regarding gradient interference and justifying the two-stage training strategy.

### 4.4. Parameter Sensitivity

A key feature of the NNEKF is batched parallel inference, which accelerates detection by segmenting the input time series into independent batches initialized via the observation mapping h(·). We investigate the impact of segment count on inference time and F1-score.

**Figure 8:**
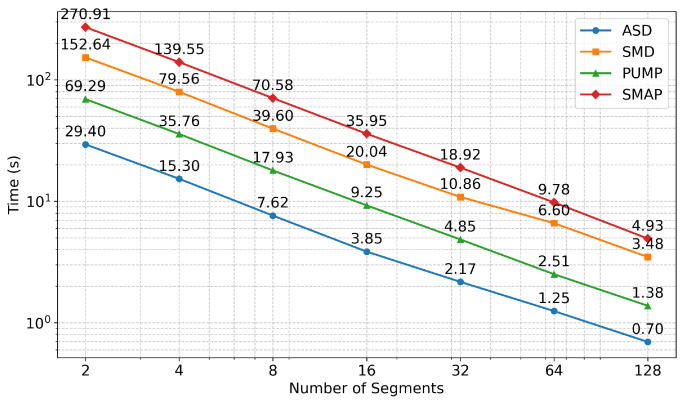
Inference time as a function of the number of parallel batches across four datasets.

As shown in Figure 8, inference time decreases exponentially with the number of segments, empirically confirming the $O(\left(T/B\right)\cdot \left({ln}^{2}+nm+m^{2}\right))$ complexity derived in Section 3.4. This near-linear speedup is crucial for real-time CPS deployments.

**Figure 9:**
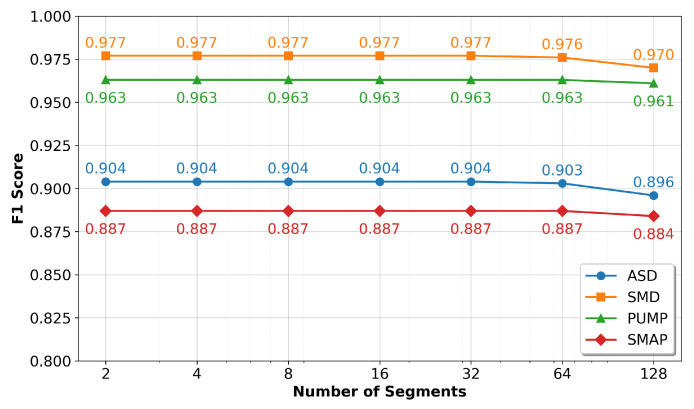
F1-score as a function of the number of parallel batches across four datasets.

Figure 9 demonstrates the robustness of our parallelization strategy: F1-scores remain stable across segment counts, with only minimal degradation even at 128 segments. This negligible decline is far outweighed by the dramatic inference time reduction—over two orders of magnitude compared to the sequential NSIBF baseline. We therefore select 32 segments as the default configuration to balance speed and accuracy.

**Figure 10:**
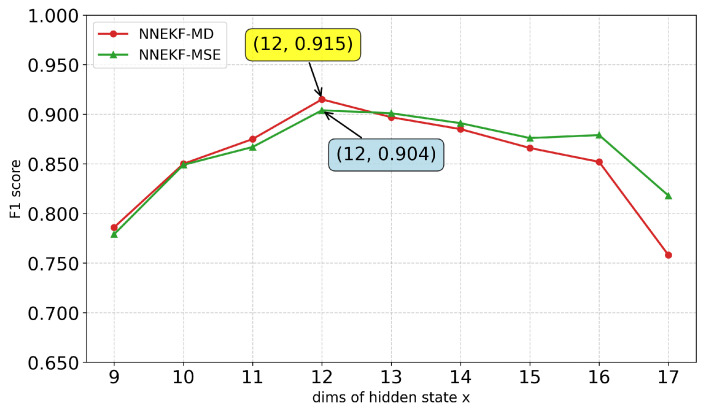
F1-scores for different state dimensions on ASD dataset.

**Figure 11:**
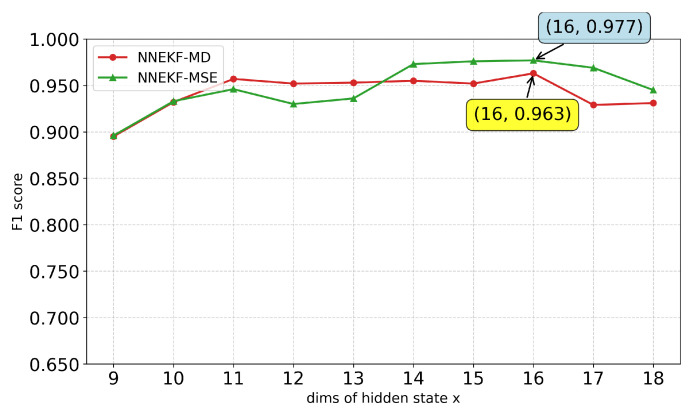
F1-scores for different state dimensions on SMD dataset.

**Figure 12:**
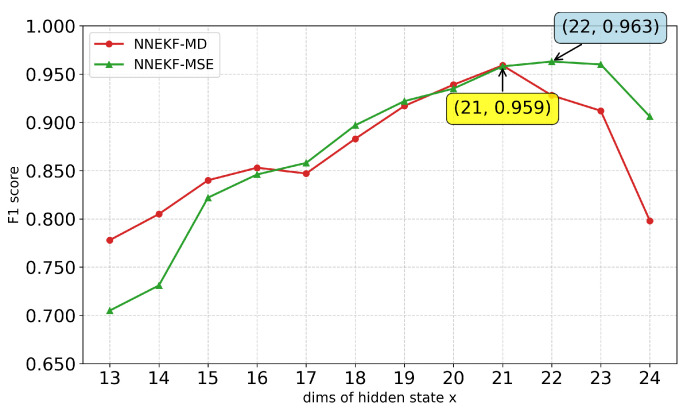
F1-scores for different state dimensions on pump dataset.

**Figure 13:**
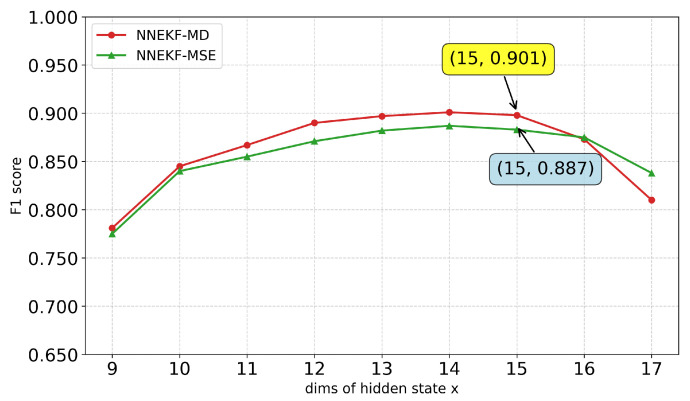
F1-scores for different state dimensions on SMAP dataset.

We further study the impact of the hidden state dimension $x_{t}$ on model performance. As shown in Figure 10, Figure 11, Figure 12 and Figure 13, a consistent trend emerges across all datasets: low-dimensional states lack sufficient capacity to encode observational information, whereas excessively high dimensions introduce redundancy and overfitting. Specifically, for ASD (observation dimension 19), the optimal state dimension is 12; for SMD (observation dimension 38), the optimal dimension is 16; for smap (observation dimension 25), the optimal dimension is 15; and for PUMP (observation dimension 44), the optimal dimension lies between 21 and 22. This pattern suggests that a moderate latent dimensionality strikes the optimal balance between representational capacity and generalization, avoiding both underfitting and the curse of dimensionality.

### 4.5. Robustness Evaluation

We evaluate the robustness of our proposed model against two prevalent types of data corruption: additive Gaussian noise and random missing values.

**Figure 14:**
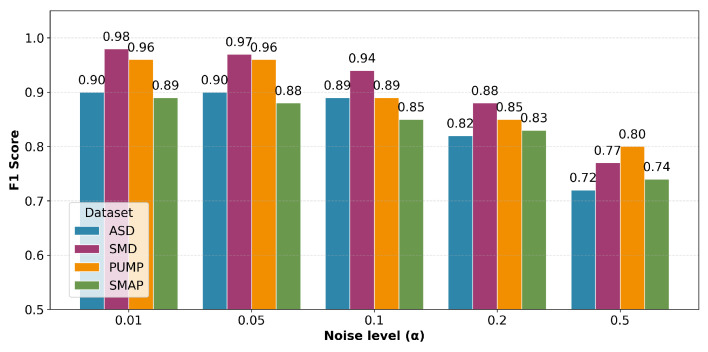
F1-scores for different noise levels across four datasets.

We inject zero-mean Gaussian noise into the input features. The noise standard deviation is scaled relative to the data range as σ=α·(max(x)−min(x)), where α denotes the relative noise intensity. We vary α across {0.01,0.05,0.1,0.2,0.5}. Figure 14 presents the F1-scores under varying noise levels. As expected, the performance degrades gradually with increasing α; however, our model maintains a competitive F1-score above 0.7 even under severe noise conditions (α=0.5).

**Figure 15:**
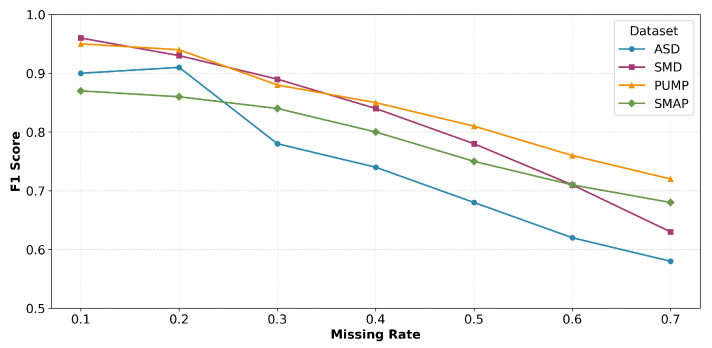
F1-scores for different missing rates across four datasets.

We further evaluate the model’s tolerance to incomplete data by randomly setting a fraction of input features to zero. The missing rate *r* varies from 0.1 to 0.7 with a step size of 0.1. Figure 15 illustrates the results. Notably, although performance declines as *r* increases, the model retains reasonable performance even when 70% of features are missing (r=0.7), demonstrating strong robustness against data incompleteness.

## 5. Conclusions

This paper proposes a Neural Network-Enhanced Kalman Filter (NNEKF), a novel anomaly detection approach for cyber-physical systems. The method integrates a neural network with a Kalman filter to learn system dynamics and optimize state estimation, achieving robust and efficient detection. Extensive experiments on four real-world CPS benchmark datasets demonstrate that the NNEKF achieves an average F1-score of 0.935, which is comparable to state-of-the-art methods, while offering low inference latency and minimal memory footprint, rendering it ideally suited for resource-constrained CPS environments.

Beyond detection accuracy, the NNEKF is designed to facilitate practical deployment in real-world applications. Latency: Under batched parallel inference, the per-step computational complexity is reduced to $O(\left(T/B\right)\cdot \left(ln^{2}+nm+m^{2}\right))$, achieving inference times that are orders of magnitude faster than those of baseline methods. Scalability: The model consistently delivers strong performance across datasets of varying sizes and dimensions (ASD, SMD, PUMP, SMAP), demonstrating its adaptability to different scales of cyber-physical systems. Online deployment: With a minimal model footprint and low inference latency, the NNEKF can be effectively deployed for online sensor monitoring and real-time alerting.

Going forward, a promising direction to address the covariance limitation is to estimate $R_{t}$ online through low-rank approximation techniques. Recent advances such as RRKF [37] and LoKO [38] demonstrate that covariance matrices can be maintained with quadratic or linear complexity. Integrating these methods into our K-network would enable lightweight online adaptation for real-time CPS deployment, enhancing robustness while preserving minimal memory footprint.

## Figures and Tables

**Table 1:** Dataset statistical information.

	Train	Test	Subsets	Dims	Anomalies (%)
ASD	102,331	51,840	12	19	4.61
SMD	304,168	304,174	12	38	5.84
PUMP	76,901	143,401	1	44	10.05
SMAP	135,183	427,617	1	25	13.13

**Table 2:** Experimental results on four datasets (ASD, SMD, PUMP, SMAP). Results are reported as mean ± half-width of 95% confidence interval over 5 independent runs. Best result is highlighted in bold.

Method	ASD			SMD		
	P	R	F1	P	R	F1
iForest	0.263±0.010	0.517±0.012	0.349±0.009	0.338±0.011	0.576±0.014	0.426±0.009
DAGMM	0.621±0.012	0.908±0.010	0.738±0.009	0.592±0.019	0.959±0.009	0.732±0.014
OmniAnomaly	0.743±0.015	0.918±0.012	0.821±0.010	0.872±0.016	0.969±0.011	0.918±0.010
AnomalyTrans	0.810±0.016	0.625±0.019	0.706±0.014	0.893±0.012	0.966±0.019	0.928±0.011
DCdetector	0.745±0.027	0.627±0.017	0.681±0.016	0.950±0.022	0.883±0.016	0.915±0.012
DADA	0.733±0.016	0.950±0.020	0.827±0.012	0.936±0.016	0.941±0.017	0.938±0.011
KAN-AD	0.839±0.019	0.926±0.012	0.880±0.011	0.956±0.014	0.980±0.012	0.968±0.010
NSIBF	0.847±0.011	0.951±0.014	0.896±0.009	0.970±0.007	0.976±0.011	0.973±0.007
NNEKF-MSE	0.881±0.019	0.929±0.014	0.904±0.012	0.988±0.009	0.967±0.017	0.977±0.010
NNEKF-MD	0.902±0.009	0.928±0.017	0.915±0.010	0.976±0.014	0.950±0.011	0.963±0.009
**Method**	**PUMP**			**SMAP**		
	**P**	**R**	**F1**	**P**	**R**	**F1**
iForest	0.922±0.011	0.475±0.007	0.627±0.006	0.431±0.014	0.520±0.019	0.471±0.011
DAGMM	0.928±0.009	0.767±0.014	0.840±0.009	0.806±0.019	0.871±0.012	0.837±0.011
OmniAnomaly	0.939±0.007	0.815±0.015	0.873±0.009	0.818±0.016	0.894±0.019	0.854±0.012
AnomalyTrans	0.944±0.016	0.987±0.012	0.965±0.010	0.822±0.011	0.979±0.019	0.894±0.010
DCdetector	0.943±0.014	0.982±0.019	0.962±0.012	0.733±0.021	0.901±0.019	0.808±0.015
DADA	0.919±0.012	0.962±0.014	0.940±0.009	0.688±0.016	0.902±0.020	0.781±0.012
KAN-AD	0.964±0.012	0.971±0.011	0.967±0.009	0.979±0.011	0.901±0.016	0.938±0.010
NSIBF	0.960±0.010	0.941±0.009	0.950±0.006	0.927±0.011	0.880±0.012	0.852±0.009
NNEKF-MSE	0.957±0.009	0.969±0.012	0.963±0.007	0.879±0.015	0.895±0.012	0.887±0.010
NNEKF-MD	0.909±0.010	0.985±0.006	0.959±0.005	0.913±0.010	0.890±0.016	0.901±0.010

**Table 3:** F1-score and inference time comparison on four datasets (ASD, SMD, PUMP, SMAP). Results are reported as mean ± half-width of 95% confidence interval over 5 independent runs. Best result is highlighted in bold, and second best is underlined.

Methods	F1-Score	Inference Time (s)			
	Avg F1	ASD	SMD	PUMP	SMAP
Baseline					
AnomalyTrans	0.873	31.81±1.43	83.90±2.56	69.92±1.96	108.42±3.07
DCdetector	0.842	8642.30±51.74	16,732.76 ± 79.58	12,718.96 ± 65.01	21,575.11 ± 110.97
DADA	0.871	15.80±0.70	76.19±2.28	45.61±1.37	102.58±2.89
KAN-AD	0.938	6.36±0.38	30.16±1.73	17.61±1.12	37.16±1.96
NSIBF	0.918	636.36±5.72	3908.17±31.24	1861.13±16.17	7239.29±63.59
Serial Inference					
NNEKF-MSE	0.933	61.47±4.51	362.92±27.23	178.18±10.42	694.94±40.61
NNEKF-MD	$\underline{0.935}$	64.80±4.59	375.14±29.32	188.05±10.58	735.02±41.61
Parallel Inference					
NNEKF-MSE	0.933	2.17±0.19	10.86±0.47	4.85±0.32	18.92±1.28
NNEKF-MD	$\underline{0.935}$	$\underline{2.23\pm 0.22}$	$\underline{11.36\pm 0.48}$	$\underline{4.97\pm 0.30}$	$\underline{19.37\pm 1.13}$

**Table 4:** Computational cost and model storage of NSIBF across four datasets.

Dataset	Training Time (s)	Inference Time (s)	Total Time (s)	Model Storage (MB)
ASD	48.20	636.36	684.56	0.43
SMD	144.75	3908.17	4052.92	0.45
PUMP	35.79	1861.13	1896.92	1.83
SMAP	61.83	7239.29	7301.12	0.49

**Table 5:** Computational cost and model storage of NNEKF across four datasets.

Dataset	Stage 1 (s)	Stage 2 (s)	Inference Time (s)	Total Time (s)	Model Storage (MB)
ASD	51.16	166.83	2.17	217.99	0.80
SMD	155.61	506.57	10.86	662.18	0.87
PUMP	40.89	132.17	4.85	177.86	3.22
SMAP	72.65	235.51	18.92	327.08	1.02

## Data Availability

The ASD and SMD datasets used in this study are publicly available and can be accessed at https://github.com/zhhlee/InterFusion/tree/main/data, accessed on 12 February 2026. The PUMP dataset used in this study is publicly available and can be accessed at https://github.com/cfeng783/NSIBF/tree/main/datasets, accessed on 12 February 2026. The SMAP dataset used in this study is publicly available and can be accessed at https://www.kaggle.com/datasets/patrickfleith/nasa-anomaly-detection-dataset-smap-msl/data, accessed on 12 February 2026. No new data were created.

## Appendix A. Hyperparameter Configuration

This appendix provides detailed hyperparameter settings for the proposed NNEKF framework, including the neural network architectures, training configurations, and inference parameters. All experiments were conducted on a virtual GPU (vGPU). The underlying physical hardware is an NVIDIA GeForce RTX 4090 with 48 GB memory.

**Table A1:** Hyperparameter settings for the NNEKF framework.

Hyperparameter	Dataset	Value
Hidden state dimension $x_{t}$	ASD	12
	SMD	16
	PUMP	21/22
	SMAP	15
Input window length *l*	ASD	10
	SMD	10
	PUMP	15
	SMAP	10
Weighting hyperparameter $w_{1}$	ALL	0.45
Weighting hyperparameter $w_{2}$	ALL	0.45
Weighting hyperparameter $w_{3}$	ALL	0.1
Hidden dims for *g* net	ASD	64
	SMD	64
	PUMP	128
	SMAP	64
Hidden dims for *h* net	ASD	64
	SMD	64
	PUMP	128
	SMAP	64
Hidden dims for *f* net	ASD	128
	SMD	128
	PUMP	227
	SMAP	150
*K*-network attention heads	ASD	4
	SMD	4
	PUMP	4
	SMAP	4
*K*-network embedding dimension	ASD	95
	SMD	190
	PUMP	220
	SMAP	150
*K*-network GRU layers	ALL	3
Learning rate (Stage 1)	ALL	$5\times 10^{-4}$
Learning rate (Stage 2)	ALL	$1\times 10^{-3}$
Epochs (Stage 1)	ALL	100
Epochs (Stage 2)	ALL	100
Parallel batches *B*	ALL	32

## References

[1] El-Shafeiy E., Alsabaan M., Ibrahem M.I., Elwahsh H. (2023). Real-time anomaly detection for water quality sensor monitoring based on multivariate deep learning technique. Sensors.

[2] Xing W., Shen J. (2024). Security control of cyber–physical systems under cyber attacks: A survey. Sensors.

[3] Blázquez-García A., Conde A., Mori U., Lozano J.A. (2021). A Review on Outlier/Anomaly Detection in Time Series Data. ACM Comput. Surv..

[4] Zamanzadeh Darban Z., Webb G.I., Pan S., Aggarwal C., Salehi M. (2024). Deep Learning for Time Series Anomaly Detection: A Survey. ACM Comput. Surv..

[5] DeMedeiros K., Hendawi A., Alvarez M. (2023). A survey of AI-based anomaly detection in IoT and sensor networks. Sensors.

[6] Khodarahmi M., Maihami V. (2023). A review on Kalman filter models. Arch. Comput. Methods Eng..

[7] Li Q., Li R., Ji K., Dai W. (2015). Kalman Filter and Its Application. Proceedings of the 2015 8th International Conference on Intelligent Networks and Intelligent Systems (ICINIS).

[8] Giraldo J., Urbina D., Cardenas A., Valente J., Faisal M., Ruths J., Tippenhauer N.O., Sandberg H., Candell R. (2019). A Survey of Physics-Based Attack Detection in Cyber-Physical Systems. ACM Comput. Surv..

[9] Kalman R.E., Bucy R.S. (1961). New Results in Linear Filtering and Prediction Theory. J. Basic Eng..

[10] Zong B., Song Q., Min M.R., Cheng W., Lumezanu C., Cho D., Chen H. (2018). Deep Autoencoding Gaussian Mixture Model for Unsupervised Anomaly Detection. International Conference on Learning Representations (ICLR 2018).

[11] Su Y., Zhao Y., Niu C., Liu R., Sun W., Pei D. Robust Anomaly Detection for Multivariate Time Series through Stochastic Recurrent Neural Network. Proceedings of the 25th ACM SIGKDD International Conference on Knowledge Discovery & Data Mining.

[12] Audibert J., Michiardi P., Guyard F., Marti S., Zuluaga M.A. Usad: Unsupervised anomaly detection on multivariate time series. Proceedings of the 26th ACM SIGKDD International Conference on Knowledge Discovery & Data Mining.

[13] Deng A., Hooi B. (2021). Graph neural network-based anomaly detection in multivariate time series. Proceedings of the AAAI Conference on Artificial Intelligence.

[14] Wang S., Zhao C., Liu X., Ni X., Chen X., Gao X., Sun L. (2025). Hybrid Deep Learning Framework for Anomaly Detection in Power Plant Systems. Algorithms.

[15] Xu J., Wu H., Wang J., Long M. (2022). Anomaly Transformer: Time Series Anomaly Detection with Association Discrepancy. arXiv.

[16] Kumar D., Addula S.R., Lind M., Brown S., Odion S. (2026). AI-Driven Hybrid Deep Learning and Swarm Intelligence for Predictive Maintenance of Smart Manufacturing Robots in Industry 4.0. Electronics.

[17] Julier S.J., Uhlmann J.K. (1997). New extension of the Kalman filter to nonlinear systems. Proceedings of the Signal Processing, Sensor Fusion, and Target Recognition VI.

[18] Djuric P., Kotecha J., Zhang J., Huang Y., Ghirmai T., Bugallo M., Miguez J. (2003). Particle Filtering. IEEE Signal Process. Mag..

[19] Revach G., Shlezinger N., Ni X., Escoriza A.L., van Sloun R.J.G., Eldar Y.C. (2022). KalmanNet: Neural Network Aided Kalman Filtering for Partially Known Dynamics. IEEE Trans. Signal Process..

[20] Choi G., Park J., Shlezinger N., Eldar Y.C., Lee N. (2023). Split-KalmanNet: A Robust Model-Based Deep Learning Approach for State Estimation. IEEE Trans. Veh. Technol..

[21] Laufer-Goldshtein B., Talmon R., Gannot S. (2018). A Hybrid Approach for Speaker Tracking Based on TDOA and Data-Driven Models. IEEE/ACM Trans. Audio Speech Lang. Process..

[22] Zhou L., Luo Z., Shen T., Zhang J., Zhen M., Yao Y., Fang T., Quan L. KFNet: Learning Temporal Camera Relocalization Using Kalman Filtering. Proceedings of the 2020 IEEE/CVF Conference on Computer Vision and Pattern Recognition (CVPR).

[23] Yoshida W., Hirose K. (2024). Fast same-step forecast in SUTSE model and its theoretical properties. Comput. Stat. Data Anal..

[24] Rangapuram S.S., Seeger M.W., Gasthaus J., Stella L., Wang Y., Januschowski T. (2018). Deep State Space Models for Time Series Forecasting. Proceedings of the Advances in Neural Information Processing Systems.

[25] Tian Y., Lai R., Li X., Xiang L., Tian J. (2020). A Combined Method for State-of-Charge Estimation for Lithium-Ion Batteries Using a Long Short-Term Memory Network and an Adaptive Cubature Kalman Filter. Appl. Energy.

[26] Ma X., Zhang S., Tang T., Yu D., Wang X., Zhang H., Ding L., Dai K. (2024). A Lightweight High-Impact Acceleration State Reconstruction Method for Multibody Dynamic Systems by an Extended Kalman Filter- Aided Time Neural Network. IEEE Sens. J..

[27] Feng C., Tian P. Time Series Anomaly Detection for Cyber-Physical Systems via Neural System Identification and Bayesian Filtering. Proceedings of the 27th ACM SIGKDD Conference on Knowledge Discovery & Data Mining.

[28] Hochreiter S., Schmidhuber J. (1997). Long Short-Term Memory. Neural Comput..

[29] Krishnapriyan A., Gholami A., Zhe S., Kirby R., Mahoney M.W. (2021). Characterizing possible failure modes in physics-informed neural networks. Advances in Neural Information Processing Systems.

[30] De Maesschalck R., Jouan-Rimbaud D., Massart D. (2000). The Mahalanobis Distance. Chemom. Intell. Lab. Syst..

[31] Li Z., Zhao Y., Han J., Su Y., Jiao R., Wen X., Pei D. Multivariate Time Series Anomaly Detection and Interpretation Using Hierarchical Inter-Metric and Temporal Embedding. Proceedings of the 27th ACM SIGKDD Conference on Knowledge Discovery & Data Mining.

[32] Hundman K., Constantinou V., Laporte C., Colwell I., Soderstrom T. Detecting spacecraft anomalies using lstms and nonparametric dynamic thresholding. Proceedings of the 24th ACM SIGKDD International Conference on Knowledge Discovery & Data Mining.

[33] Liu F.T., Ting K.M., Zhou Z.H. (2008). Isolation Forest. Proceedings of the 2008 Eighth IEEE International Conference on Data Mining.

[34] Yang Y., Zhang C., Zhou T., Wen Q., Sun L. DCdetector: Dual Attention Contrastive Representation Learning for Time Series Anomaly Detection. Proceedings of the 29th ACM SIGKDD Conference on Knowledge Discovery and Data Mining.

[35] Shentu Q., Li B., Zhao K., Shu Y., Rao Z., Pan L., Yang B., Guo C. Towards a General Time Series Anomaly Detector with Adaptive Bottlenecks and Dual Adversarial Decoders. Proceedings of the 13th International Conference on Learning Representations, ICLR 2025.

[36] Zhou Q., Pei C., Sun F., Jing H., Gao Z., Zhang H., Xie G., Pei D., Li J. KAN-AD: Time Series Anomaly Detection with Kolmogorov–Arnold Networks. Proceedings of the International Conference on Machine Learning.

[37] Schmidt J., Hennig P., Nick J., Tronarp F. (2023). The rank-reduced Kalman filter: Approximate dynamical-low-rank filtering in high dimensions. Advances in Neural Information Processing Systems.

[38] Abdi H., Sun M., Zhang A., Kaski S., Pan W. (2024). LoKO: Low-Rank Kalman Optimizer for Online Fine-Tuning of Large Models. arXiv.

